# Fungemia caused by *Aureobasidium pullulans* in a patient with advanced AIDS: a case report and review of the medical literature

**DOI:** 10.1099/jmmcr.0.005144

**Published:** 2018-03-14

**Authors:** Jaimie Mittal, Wendy A. Szymczak, Liise-anne Pirofski, Benjamin T. Galen

**Affiliations:** ^1^​ Department of Internal Medicine, Division of Infectious Diseases, Albert Einstein College of Medicine and Montefiore Medical Center, 3411 Wayne Ave., Bronx, NY 10467, USA; ^2^​ Department of Pathology, Albert Einstein College of Medicine and Montefiore Medical Center, 111 E. 210th Street, Bronx, NY 10467, USA; ^3^​ Department of Internal Medicine, Division of Hospital Medicine, Albert Einstein College of Medicine and Montefiore Medical Center, 1825 Eastchester Rd., Bronx, NY 10461, USA

**Keywords:** AIDS, fungaemia, *Aureobasidium pullulans*, micafungin

## Abstract

**Introduction:**

*Aureobasidium pullulans* is a dematiaceous, yeast-like fungus that is ubiquitous in nature and can colonize human hair and skin. It has been implicated clinically as causing skin and soft tissue infections, meningitis, splenic abscesses and peritonitis. We present, to our knowledge, the second case of isolation of this organism in a patient with AIDS along with a review of the literature on human infection with *A. pullulans*.

**Case presentation:**

A 49-year-old man with advanced AIDS and a history of recurrent oesophageal candidiasis was admitted with nausea with vomiting, and odynophagia. He was treated as having a recurrence of oesophageal candidiasis. Given prior *Candida albicans* isolate susceptibilities and chronic suppression with fluconazole, he was started on micafungin with eventual improvement in his symptoms. A positive blood culture from admission was initially reported to be growing yeast, but four days later the isolate was recognized as a dematiaceous fungus. The final identification of *A. pullulans* was not available until 1 month after admission. He had completed a 3-week course of micafungin prior to the identification of the isolate, and repeat cultures were negative.

**Conclusion:**

*A. pullulans* fungemia is rare but can occur in patients with immune suppression or indwelling catheters. The significance of isolating *A. pullulans* from a blood culture in terms of whether it is the causative agent of a state of disease often cannot be determined because skin colonization is possible. Further work is needed to clarify the clinical implications of *A. pullulans* fungemia.

## Introduction

*Aureobasidium pullulans* is a dematiaceous yeast-like fungus [1]. It produces melanin in its cell wall giving its colonies their characteristic black appearance after a few days on culture medium [1, 2]. *A. pullulans* is found in soil, rocks, wood and household dust and can colonize human hair and skin [3]. It may be considered a contaminant when isolated from an immunocompetent host unless it is isolated from a sterile site, found in multiple specimens or isolated in the setting of clinical presentation consistent with infection [4]. Like other saprophytic fungi, *A. pullulans* can cause disease in the setting of immunocompromised conditions [5]. In such patients, it has been implicated in skin and soft tissue infections, meningitis, splenic abscesses and peritonitis [6]. Clinically significant fungaemia with *A. pullulans* is a rare occurrence. There are case reports, and infections are often associated with indwelling devices and prolonged hospital exposure. Our knowledge of the clinical presentation of *A. pullulans* infection, potential sources of invasive disease and standards of treatment remain limited. Here, we present a case of a positive blood culture isolation of *A. pullulans* in an AIDS patient, whereby the need for molecular identification delayed diagnosis and limited the timely evaluation for a source. Given that only one prior case of *A. pullulans* fungemia has been reported in a patient with AIDS, this case illustrates the difficulty in determining its significance and further management.

## Case report

**Fig. 1.**
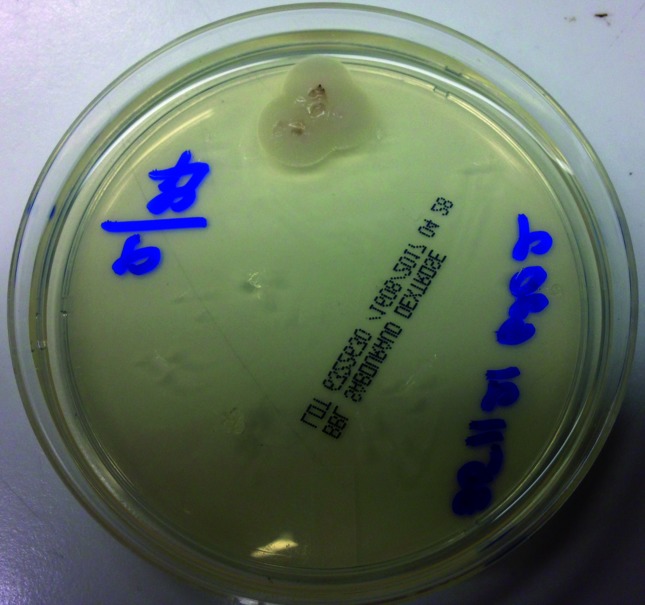
*A. pullulans* colony appearance by day 4 on repeated sub-culture of the isolate.

**Fig. 2.**
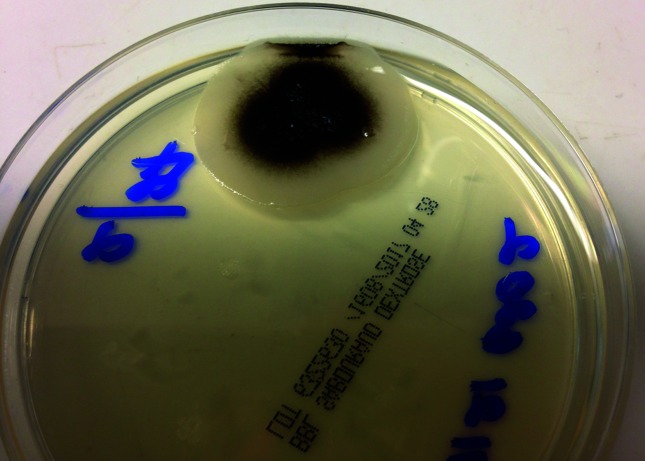
*A. pullulans* colony appearance by day 7 on repeated sub-culture of the isolate.

A 49-year-old man with advanced AIDS (CD4 8 cells µl^−1^) and overall poor performance status on antiretroviral therapy was admitted in November 2016 with 10 days of nausea with vomiting and reported retching, chest pain, odynophagia and persistent diarrhoea. He had been admitted to the hospital at least five times in the previous 2 years and had a history of azole-resistant oral and esophageal candidiasis. Over the preceding months he was on suppressive therapy with fluconazole as an outpatient. His examination on admission was noteworthy for cachexia and oral thrush. He was started on micafungin 100 mg daily with delayed improvement in his upper gastrointestinal symptoms by day 5. He was awaiting discharge with a planned three-week course of micafungin. However, on day 7, aerobic blood culture collected on admission showed growth and Gram stain was reported as budding yeast. Given his clinical improvement, lack of fever and no new complaints, he was continued on micafungin with further diagnostics ordered. Ophthalmologic exam, echocardiogram, computerized axial tomography imaging of his head, lumbar puncture and cryptococcal antigen were all negative. Upper endoscopy was not done as his symptoms improved. Repeat fungal blood cultures were collected on day 7, which later showed no growth. The budding yeast were not identified as a possible dematiaceous fungus until hospital day 11 (Figs 1 and 2). Multiple colonies were seen on the initial growth media, notably black in color by this time; however, complete identification could not be made by morphology alone and the isolate was sent to the Department of Health. Given the patient’s improvement in his presenting symptoms, he was discharged home to complete a final week of micafungin with planned follow up as an outpatient.

The isolate was eventually identified as *A. pullulans* by ribosomal RNA sequencing, and results were released 1 month after the patient’s admission date. Based on the report from the Department of Health using the Clinical and Laboratory Standards Institute M38-A2 reference method, the isolate of *A. pullulans* from our patient was tested against amphotericin B, caspofungin, micafungin, posaconazole, fluconazole and voriconazole (Table 1). There are no interpretation standards for susceptibility to any of these drugs. Since our initial encounter, the patient returned to our facility two times in the span of 5 months with recurrence of his symptoms and progression of his poor performance status. On these admissions, he had repeat blood cultures, but this fungus was not recovered. He also had repeat upper endoscopy and fungal hyphae were seen on biopsy with Periodic acid–Schiff-D stain. Unfortunately, no cultures or additional staining procedures were done to identify them as the thought was that the organisms on biopsy were consistent with a clinical picture of recurrent candida esophagitis. The biopsy specimens was reviewed while writing this report, and a Fontana-Mason stain was performed to determine if these fungal elements reported previously contained melanin in their cell walls. However, the fungal elements were not well visualized on inspection and the stain did not highlight any melanin-containing elements. The procedure was largely inconclusive. The patient ultimately died of respiratory failure on his second readmission, 5 months after our encounter.

**Table 1.** Susceptibility testing of the *A. pullulans* isolate by the Department of Health

Anti-fungal agent*	MIC
Amphotericin B	0.12 µg ml^−1^
Caspofungin	<0.015 µg ml^−1^
Fluconazole	8.0 µg ml^−1^
Micafungin	0.25 µg ml^−1^
Posaconazole	0.015 µg ml^−1^
Voriconazole	0.12 µg ml^−1^

MIC, minimum inhibitory concentration.

*Testing done for study purposes only, no standard for interpretation.

## Discussion

Invasive infection with *A. pullulans* has been reported previously, but only one other case has been described in a patient with AIDS (Table 2) [7]. All reported cases occurred in patients with conditions such as cancer, autoimmune diseases and bone marrow transplant, or in patients with significant hospital exposure. In these reports, symptoms at presentation were mostly non-specific and usually included fevers. In some reports, including the one other patient with AIDS, the organism was initially identified as a yeast-like organism. As in the case described above, identification of this fungus was unexpected, made after observation of certain growth characteristics on medium, and then required molecular confirmation.

**Table 2.** Summary of case reports of bloodstream infections with *A. pullulans*

Author, year, reference	Source	Age, Sex	Comorbidities	Clinical background and sample collection	Final identification method	Treatment	Outcome
Mehta *et al*. (2017) [5]	Hickman catheter	66, M	-Single kidney -TPN-dependent -Chron’s Disease	Presented with weeks of fever and isolated *Staphylococcus* bacteraemia initially; repeat cultures were collected through the patient’s catheter	-MALDI-TOF -DNA sequencing	Micafungin; amphotericin B	Survived
Huang *et al*. (2008) [1] (Case 1)	CVC	61, F	-Hepatocellular carcinoma	Presented with altered mental status and found to have intracranial metastatic disease; had 26-day admission with CVC in place and developed fever; catheter tip and blood cultures drawn initially identified *Cryptococcus laurentii* until black colonies were seen on culture plates	-Fluorescence-based technology in combination with culture characteristics	Catheter removal	Survived
Huang *et al.* (2008) [1] (Case 2)	CVC	54, F	-Pleural cutaneous fistula -Empyema -Oesophageal fistula	Presented for complicated empyema; 54 days into admission had a fever and catheter site had erythema present; fungal blood cultures and catheter tip were sent for culture	-ITS sequencing	Amphotericin B; fluconazole	Survived
Girardi *et al.* (1993) [10]	CVC	53, F	-Stage 3 ovarian cancer -TPN-dependent	Presented with fevers, blood cultures had Gram-negative organisms present; day 13 patient had fever but no erythema seen at the catheter site; cultures that were taken 5 days before this fever showed yeast identified as *Trichosporon* by biochemical testing; isolate was sent to the state lab for further testing	-Not specified	Amphotericin B	Died
van Hougehhouck-Tulleken *et al*. (2016) [7]	Septic arthritis	28, F	-AIDS (CD4 168)	Presented with 4-month history of oligoarthritis and rash, osteomyelitis found on images of the talus and tibia; cultures were taken of synovial fluid, blood and tissue specimens; organism was initially identified as *Cryptococcus neoformans* b*y* colorimetric testing kits until repeat testing was done	-Phenotypic testing of synovial fluid -ITS sequencing of blood and synovial fluid	Amphotericin B; fluconazole	Survived
Bolignano *et al.* (2003) [2]	CVC, open wounds or SSI	28, M	-TPN-dependent -Prolonged intubation	Admitted after head trauma from car accident; owing to persistent fevers, repeat cultures were collected peripherally and from his CVC	-Culture growth and appearance characteristics	Fluconazole	Survived
Hawkes *et al*. (2005) [11]	CVC	4 months, M	-None	Mother presented in labour with complicated delivery and course with emergent cardiac surgery and cardiac collapse; patient had CVC in place and cultures sent from the catheter on day 86 and 92 of admission; tissue on autopsy was also sent from the pulmonary arteries	-Colony morphology and microscopic appearance	Amphotericin B	Died
Mershon-Shier *et al.* (2011) [12]	CVC	11, M	-Intestinal lymphangiectasis -Lymphopaenia -Protein losing enteropathy	He was admitted with fever and concern for infection of his previously placed port; cultures collected from his CVC on admission were positive; catheter tip was later sent for culture and was negative	-Phenotypic testing	Amphotericin B	Survived
Joshi *et al*. (2010) [6]	CVC	11, M	-Bone Marrow transplant recipient	Admitted for second transplant and on day 0 developed skin rash and fevers; blood cultures were collected as well as skin biopsy and initial identification of the organism present was *Candida*	-Culture growth and appearance characteristics	Amphotericin B; voriconazole	Survived
Kaczmarski *et al.* (1986) [13]	Hickman catheter	28, M	-Acute myeloid leukaemia	Patient was undergoing induction chemotherapy when he developed fever and altered mental status; cultures were collected peripherally and from his Hickman catheter and sent to a reference lab for identification	-Not specified	Amphotericin B	Died

CVC, central venous catheter; ITS, internal transcribed spacer; MALDi-TOF, matrix-assisted laser desorption/ionization time-of-flight; SSI, surgical site infection; TPN, total parenteral nutrition.

*A. pullulans* has significant morphologic variability that is affected by environmental conditions, making its identification a challenge [5]. It shows optimal growth at 30 °C on potato dextrose agar [5]. As seen in Figs 1 and 2, it initially appears as creamy white colonies and 7 days from initial plating it changes to its characteristic black appearance due to melanin within the cell wall. Melanin is thought to be a virulence factor for dematiaceous fungi [8]. It is localized to the cell wall, and the exact mechanism of production is unknown. Melanin provides resistance to destructive agents such as free radicals, toxic metals and ionizing radiation [8]. It is proposed as providing protection from phagocytic cells in this way. It is also thought to be able to bind hydrolytic drugs such as some antifungals and not allow their access to the cell membrane, thus rendering them ineffective [8]. It is believed to play a role in the organism’s survival and pathogenesis of disease.

Patient outcomes varied among the cases reviewed. Some patients survived a course of therapy and others died before completion, likely from their other significant medical comorbidities. Therapies ranged from prolonged courses of amphotericin B to just removal of potential sources, if identified, without antifungal therapy. Although there are no current interpretation standards for susceptibility to any antifungals, we compared our isolates minimum inhibitory concentrations (MICs) with what had been seen in a 2014 study that evaluated 104 environmental and clinical isolates [9]. For all the drugs tested, MICs for our isolate were well below the reported MICs that inhibited 90 % of *in vitro* growth in this study. The isolate in our case also had a MIC of 8.0 µg ml^−1^ for fluconazole, compared with an average MIC of 64 µg ml^−1^ needed to inhibit 50 % of growth in this *in vitro* study [9]. It is possible the patient’s extensive exposure to fluconazole as suppressive therapy for oral candidiasis led to partial treatment of an unknown source of *A. pullulans*. Our patient was treated with micafungin given the initial impression that he had azole-resistant candidiasis. Subsequent blood cultures documented clearance of the *A. pullulans* fungemia. Micafungin was also used with a good response in one other case of *A. pullulans* fungaemia (Table 1) [5].

The question that remains is how the organism gained access to our patient’s blood. He had no indwelling catheter, open wounds or rashes as seen in other case reports (Table 2). Given his presenting symptoms, an upper gastrointestinal mucosa or oropharyngeal source might have led to his fungemia. Unfortunately, endoscopy was not done initially, and he improved before his culture data was reported. It is also possible his skin was colonized and growth in his blood culture was a contaminant. However, this is difficult to determine in the setting of our patient’s advanced AIDS. A 2017 case report of *A. pullulans* fungaemia noted the use of 1,3 β-D-glucan as a marker of infection, despite repeat negative cultures in the setting of a central venous catheter. The authors treated their patient for a year with good outcome and altered the regimen with changing serum levels of this marker [5]. This case was reported after our patient expired, but it illustrates a possible diagnostic and therapeutic strategy to be considered in the future.

Fungemia with *A. pullulans* is more often seen in those that are critically ill or immunocompromised. After reviewing the literature on this organism, we conclude that evidence to guide the workup and treatment of *A. pullulans* fungemia is lacking. *A. pullulans* has only been reported one other time in a patient with AIDS. If the blood culture result with *A. pullulans* growth represented fungemia from an unknown source rather than a contaminant, it is the second time this organism was successfully cleared from the blood with micafungin but this time in a patient with advanced AIDS.
